# Real-Time Monitoring of the Formation and Culture of Hybrid Cell-Microbiomaterial Spheroids Using Non-Faradaic Electrical Impedance Spectroscopy

**DOI:** 10.1021/acsbiomaterials.5c00402

**Published:** 2025-09-18

**Authors:** Maria G. Fois, Seppe Bormans, Thijs Vandenryt, Alexander P. M. Guttenplan, Yousra Alaoui Selsouli, Clemens van Blitterswijk, Zeinab Tahmasebi Birgani, Stefan Giselbrecht, Pamela Habibović, Ronald Thoelen, Roman K. Truckenmüller

**Affiliations:** † MERLN Institute for Technology-Inspired Regenerative Technology, 5211 Maastricht University, Universiteitssingel 40, 6229 ER Maastricht, The Netherlands; ‡ Institute for Materials Research, 54496 Hasselt University, Wetenschapspark 1, 3590 Diepenbeek, Belgium

**Keywords:** micro(fluidic) bioreactors, microwell arrays, spheroids, microbiomaterials, non-Faradaic electrical impedance spectroscopy, real-time monitoring

## Abstract

Cellular spheroids are considered a popular option for modeling healthy and diseased tissues *in vitro* and as injectable therapies. The formation and culture of spheroids can make use of different three-dimensional (3D) culture platforms, but the spheroids’ analysis often has to rely on endpoint assays. In this study, we propose a microfluidic bioreactor to culture and nondestructively monitor human mesenchymal stem cell (hMSC) spheroids over time using non-Faradaic electr­(ochem)­ical impedance spectroscopy (EIS). For this, an array of porous microwells thermoformed from ion track-etched thin films and a pair of sensing electrodes from transparent indium tin oxide are integrated into the flow and culture chamber of the bioreactor. To measure the spheroid’s electrical properties, the electrodes are connected to a frequency response analyzer (FRA), with a multiplexer in between to enable the operation of more than one bioreactor at the FRA at the same time. We find differences between the complex resistance/impedance and/or capacitance data of a reference condition without cells, a two-dimensional (2D) hMSC culture, hMSC spheroids, and hybrid spheroids aggregated from hMSCs and titanium or hydroxyapatite microparticles. We also found differences between different culture durations. These results suggest that our device can sense the presence and spatial arrangement of cells and micro­(sized) biomaterials as a function of time.

## Graphical abstract

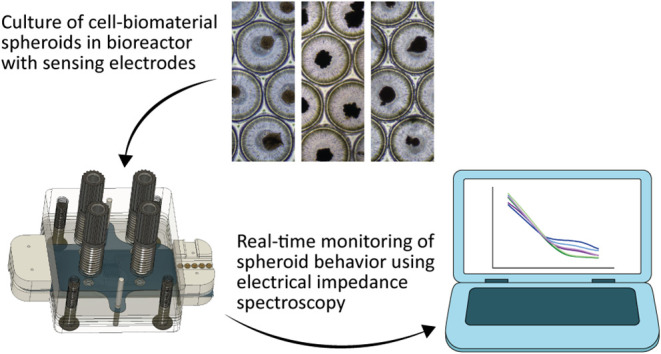

## 1 Introduction

In the context of biomedical research and development, including the field of regenerative medicine, efforts have shifted from two-dimensional (2D) to three-dimensional (3D) cell culture models, with the aim of mimicking the 3D (micro)­environment of native tissues.^1^ Among different approaches, 3D cell aggregates in the form of cell spheroids have been shown to be promising for modeling healthy and diseased tissues *in vitro*,^2,3^ injectable cell therapies,^4^ large bone defect repair,^5^ and amelioration of ischemic stroke.^6^ Other examples include the use of mesenchymal stem cell (MSC) spheroids for the regeneration of a rat calvarial bone defect,^7^ and of adipose-derived stem cell spheroids as building blocks for modeling an osteochondral tissue.^8^ Despite an increasing use of spheroids, their production and analysis remain relatively difficult and time-consuming, in comparison with conventional 2D cell culture setups.^9^ Therefore, there is a need for new production and analysis methods for cell spheroids that are facile and less labor- and time-consuming. In this context, microfabrication techniques offer interesting opportunities for establishing high-throughput culture platforms for cell spheroids. For example, spheroids have been successfully formed and cultured in cast agarose microwells^10^ and microthermoformed polycarbonate (PC) microwells^11,12^ as well as obtained using droplet-based microfluidics.^13^ The characterization of spheroids is largely performed by (endpoint-based) techniques requiring much effort and time, often associated with low yield and reproducibility.^14^ To address these challenges, recent advances in analytical methods have focused on high-content analysis, i.e., simultaneous assessment of multiple readouts, often achieved using automated microscopy,^11^ matrix-assisted laser desorption/ionization (MALDI) mass spectroscopy imaging,^15,16^ and optical coherence tomography.^17^

The development of real-time analytical techniques to monitor spheroid formation, growth, viability, and other relevant biological parameters may offer interesting advantages in terms of reducing the required number of samples while increasing the experimental time-resolution.^18^ In this context, electr­(ochem)­ical impedance spectroscopy (EIS)^19^ may be an interesting method. With this noninvasive analytical technique, a sinusoidal current or voltage is applied to a specimen over a selected frequency range to measure its impedance. The analysis of this obtained impedance spectrum provides information regarding the specimen’s electrical properties. EIS can be categorized into two variants based on the electrochemical processes occurring at the measurement electrode(s). In Faradaic EIS, charge-transfer reactions, such as redox reactions, take place at the electrolyte–electrode interface. In non-Faradaic EIS, no redox reactions occur; instead, the impedance is primarily determined by changes in the double-layer capacitance at the electrode surface.^20^ EIS has been used as a label-free sensing tool to determine the proliferation of murine-derived BV-2 microglial cells, Chinese hamster ovary (CHO) cells, and human embryonic kidney (HEK)­293T cells,^21^ the proliferation of carotid endothelial cells on different materials,^22^ and the differentiation of Saos-2 osteosarcoma cells on pyrolytic carbon electrodes.^23^ EIS has also been used to determine the barrier integrity of Caco-2 colon carcinoma cell layers under various conditions by means of transepithelial electrical resistance (TEER) analysis.^24^ And EIS has been used for the measurement of cell spheroids formed from monocultures of human fibroblasts and canine kidney epithelial cells as well as cocultures of the two cell types, where differences in the impedance signals were correlated with the different cell populations forming the spheroids.^25^ In another study, the conventional hanging-drop spheroid formation and culture technique was successfully combined with EIS measurement of cardiac spheroid size and beating frequency.^26^ To accomplish this, two pairs of microelectrodes were placed directly inside of the hanging-drop culture support. The first pair measured the drop size over time, while the second pair measured the time course of the size of the spheroid within the drop. Changes in the impedance signals were correlated with the variation of the drop and spheroid size, which were assessed by visual inspection. Similarly, for the spontaneous beating of the cardiac spheroids, each spike in the EIS signal corresponded to a contraction of the spheroid, which was verified by optical microscopy. EIS electrodes have also been integrated into microfluidic perfusion chambers for automated spheroid culture, enabling the continuous monitoring of multiple biological parameters such as spheroid growth and drug response.^27^ For this, the culture platform was tilted at fixed intervals of time. During this motion, the spheroids traveled from one chamber to another along two microelectrodes, which recorded the EIS signal between them. This signal over time was associated with spheroid growth and with growth inhibition and dissociation in the presence of the tested fluorouracil anticancer drug. This behavior was also observed optically.

In this study, we present a microfluidic bioreactor to monitor the formation and behavior of spheroids using real-time non-Faradaic EIS, from here on simply termed EIS (Figure 1). The bioreactor is designed to accommodate a microwell array, which is made from a porous polymer film/membrane and suspended in the middle of the culture chamber. There, single-cell suspensions can be dispensed and aggregated into spheroids, directly within the EIS sensing platform. The porosity of the microwells allows an ion flux through the membrane, enabling EIS monitoring of the microwells’ content. The bioreactor already allows monitoring of the stage of spheroid formation through EIS. This contrasts with many other studies where the spheroids are first preformed in an external culture dish before being collected, transferred to, and monitored in the EIS platform. The bioreactor is completely see-through, as it is made from a stack of optically transparent materials. This includes transparent measurement electrodes from indium tin oxide (ITO), and cylindrical pores of the thin-walled microwells only minimally interfering with the microscopic inspection, as opposed to, for example, more bulky porous biomaterials with cellular pore morphology.

**Figure 1:**
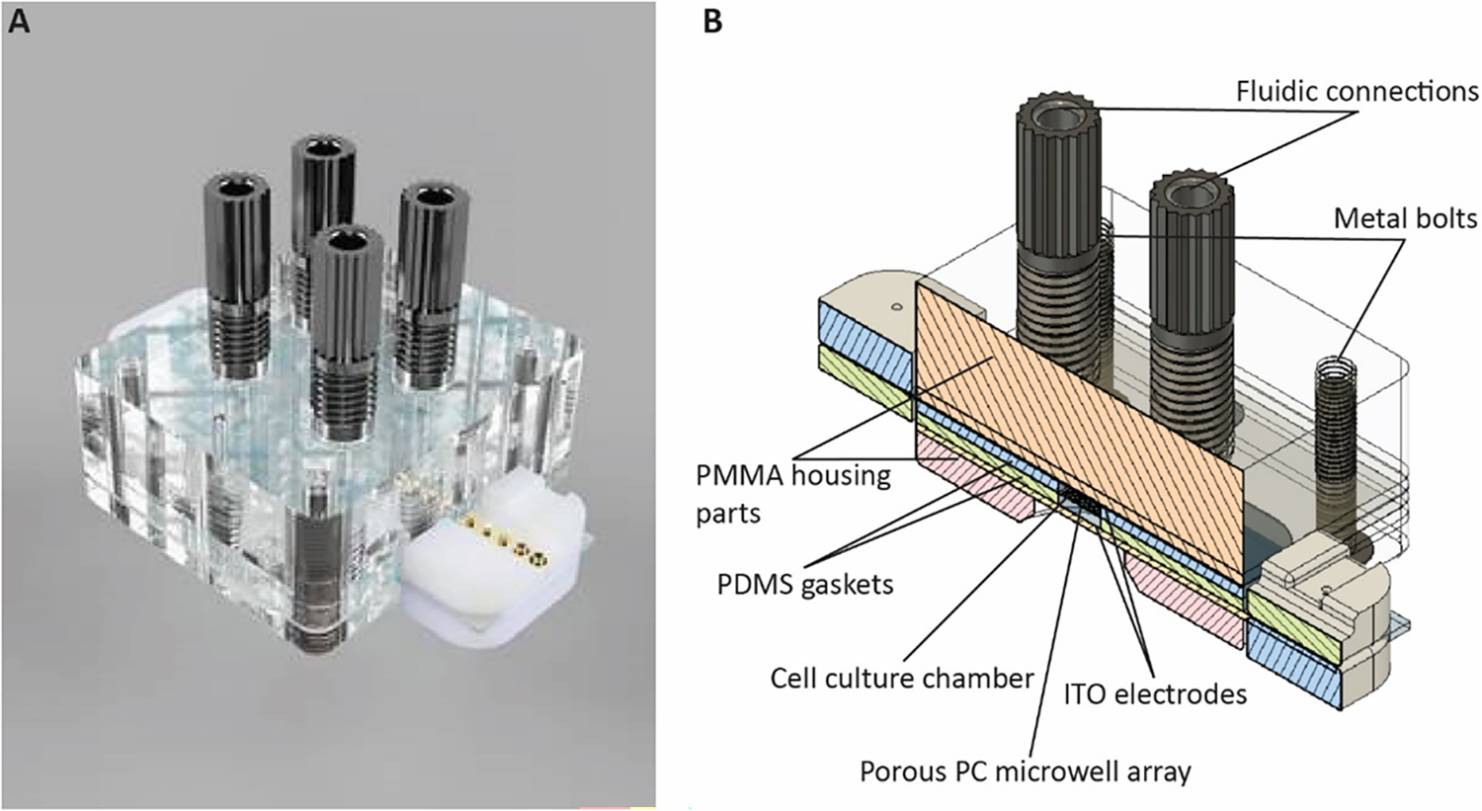
Microfluidic bioreactor. (A) Photorealistic rendering of the bioreactor. The length, width, and height of the bioreactor (without the protruding fluidic connections and electrode connectors) are 50, 45, and 18 mm, respectively. (B) Schematic representation of a cross-section of the assembled bioreactor, indicating individual components. PMMA: poly­(methyl methacrylate); PDMS: poly­(dimethylsiloxane); PC: polycarbonate; ITO: indium tin oxide. Illustrations were made using Autodesk Fusion360.

We monitored the formation of human MSC (hMSC) spheroids, with or without micro­(sized) biomaterials, by EIS, thereby also comparing it to a 2D/monolayer hMSC culture on the curved surfaces of the microwell array and the cell culture medium-only condition. The results suggest that the bioreactor may be suitable for the culture and nondisruptive monitoring of cell spheroids over time.

## 2 Experimental Section

### 2.1 Bioreactor Design, Fabrication, and Assembly

The microfluidic bioreactor consisted of a stack of multiple custom-made components (Figure 1B). Starting from the top, the first layer was a mechanically machined optically transparent housing from poly­(methyl methacrylate) (PMMA) containing four threaded holes to mount fittings (IDEX) for connecting tubing (IDEX). Underneath the PMMA housing, a laser-cut poly­(dimethylsiloxane) (PDMS) gasket (not shown in Figure 1B) was placed to seal the system and prevent leakage. Underneath the PDMS layer, a continuous electrode from ITO coated on a poly­(ethylene terephthalate) (PET) film (Sigma-Aldrich, Merck; film thickness: 5 mil; surface resistivity: 60 Ω m^–2^) was placed, which on the other side was in contact with the culture chamber of the bioreactor through another gasket from laser-cut PDMS. The film-based porous microwell array resided in the middle of the bioreactor’s culture chamber, sandwiched between two PDMS layers. Beneath the PDMS layer under the microwell array, the stack was completed by a second ITO electrode on a PET film and a second PMMA housing. The bioreactor was assembled manually with the help of two metal guide pins to ensure good alignment and fastened with four metallic bolts, one at each corner of the bioreactor. Prior to cell culture, the individual parts of the bioreactor were placed into a sealed transparent plastic bag and exposed to ultraviolet (UV) light (λ = 365 nm; UVP, CL-1000 Ultraviolet Crosslinker) for 15 min, followed by assembly inside a laminar flow hood.

### 2.2 Microwell Array Design, Fabrication, and Characterization

Porous, round-/U-bottom microwells were fabricated by proprietary microthermoforming^27^ of microporous polymer films/membranes in the form of ion track-etched films from PC, as described previously.^28,29^ The selected PC membranes (it4ip) had an initial thickness of 50 μm, a nominal pore size of 0.4 μm, and a pore density of 10^6^ pores cm^–2^. To form the microwells, a brass mold with an array of 30 micro blind-holes, hexagonally arranged in 5 rows with 6 holes each and with a diameter of 550 μm, was used. The porous film was laminated with a 50 μm-thick nonporous polypropylene (PP) film (DURABLE). Then, in a hot press (Specac), the two films were sandwiched between the brass mold and counter plate, also from brass, and including a port for pressurized nitrogen. A (gas) prepressure of 1.5 bar was applied until the heated press reached the forming temperature of 154 °C. Next, a forming pressure of 20 bar was applied to the system. At this point, the temperature was allowed to slowly decrease to 100 °C. Finally, with care, the films were demolded, and the PC film was separated from the PP film, which was discarded.

Visual inspection of the gold-sputter-coated (Quorum Technologies, SC7620) thermoformed microwells was performed using scanning electron microscopy (SEM; JEOL, JSM-IT200) at an acceleration voltage of 10 kV and a working distance of 10 mm. The microwells’ maximum outer diameter and depth (as height from the backside) and their pore size were measured using confocal laser scanning profilometry (Keyence, VK-X200).

### 2.3 Preparatory Cell Culture

hMSCs were purchased (Lonza, Cat. No. PT-2501, lot No. 19TL329433), expanded according to the manufacturer’s instructions, subcultured to passage 3, and kept in liquid nitrogen until use. For this, the cells were thawed and seeded at a density of 2500 cells cm^–2^ in a T75 tissue culture flask in a basic cell culture medium composed of α-minimum essential medium (α-MEM; Thermo Fisher Scientific, Gibco) supplemented with 10% fetal bovine serum (FBS; Sigma-Aldrich, lot No. BCBX5318), 0.2 mM 2-phosphate sesquimagnesium salt hydrate (Sigma-Aldrich), and 100 U mL^–1^ penicillin and 100 μg mL^–1^ streptomycin (Thermo Fisher Scientific, Gibco).

### 2.4 Cell Seeding into the Bioreactor

One day before the experiment, the microfluidic bioreactor was assembled, and its interior was wetted with a 70% v/v ethanol (Boom) solution in water and then rinsed twice with sterile cell culture-grade water (Cytiva, Hyclone). The wetting and rinsing steps were performed via all four fluidic ports of the bioreactor reach all of the inner surfaces. The microwell arrays were coated overnight with a 1% w/v Pluronic F108 (Sigma-Aldrich) solution in sterile cell culture-grade water by dispensing 50 μL of this solution into the bioreactor’s top compartment. The Pluronic coating prevents the adsorption of proteins and other macromolecules on the surface, in this case of the PC microwell array, consequently increasing the surface’s cell repellence,^30^ and therefore supporting the formation of cell spheroids.^12^ In the bottom compartment, sterile cell culture water was dispensed to prevent the interior of the bioreactor from drying during the coating. For the 2D culture of hMSCs in the microwells, no Pluronic coating step was performed to allow cell adhesion to the (curved) surface of the microwell array.

The next day, both the top and bottom compartments of the bioreactor were rinsed twice with basic culture medium. The hMSCs were trypsinized and resuspended in basic medium and counted. 500,000 cells in 100 μL basic medium, equally divided over two ports, were seeded into the upper compartment of the bioreactor. The cells were allowed to settle in the microwells at 37 °C and at 5% CO_2_ for about 15 min, after which the EIS measurements started. Medium refreshment occurred every day, during which the cells inside the bioreactor were also monitored using bright-field microscopy.

The average diameter of the Ti and HA microparticles (Figure S1A,B, respectively) was 25.79 ± 10.33 μm and 103.45 ± 37.39 μm,^31^ respectively. The particle size distribution was measured via image analysis in ImageJ^32^ after applying basic adjustments and subsequently making the images binary (Figure S1C,D, respectively). In the case of the formation of hybrid cell-microbiomaterial spheroids, about 5000 microparticles of (unalloyed) commercially pure titanium (Ti; AP&C) or about 200 microparticles of hydroxyapatite (HA; fabricated via droplet-based microfluidics at the MERLN institute) were resuspended in basic medium and seeded into the microwells through the ports connected to the bioreactor’s upper compartment prior to cell seeding. The different numbers of the differently sized microparticles were chosen in such a way that their surface area was roughly the same.

### 2.5 EIS Measurements

The EIS measurements were conducted using a frequency response analyzer (FRA) for E­(lectrochemical)­IS (PalmSens, PalmSens 4) in combination with a related software interface (PalmSens, PSTrace, version 5.8). The impedance measurements were performed every hour in a frequency range from 0.1 Hz to 100 kHz. The applied AC voltage was 0.01 V. No DC bias was applied. The ITO electrodes of the microfluidic bioreactor were connected to the FRA through a system of custom-made electrical connectors and a multiplexer (PalmSens, MUX8-R2). For each of two bioreactors and pairs of electrodes per culture run, one input channel of the multiplexer was used, and EIS measurements were recorded for one bioreactor after the other.

### 2.6 Quantification of Spheroid Area

The size of the spheroids cultured inside the microfluidic bioreactor was determined by image analysis of bright-field images, which were acquired daily during the culture using an inverted microscope (Nikon Instruments, Eclipse TS100). The projection area of the spheroids was measured via image analysis in ImageJ^32^ after applying basic adjustments and subsequently making the images binary.

### 2.7 Assessment of Cell and Spheroid Morphology

Fluorescence staining, followed by confocal fluorescence imaging, was performed as an endpoint analysis of the hMSC spheroids and 2D cultures within the microfluidic bioreactor. To this end, after 48 h of culture, the bioreactors were disassembled to retrieve the microwell arrays. The cells were fixed with a 10% v/v formaldehyde (Sigma-Aldrich) solution in phosphate-buffered saline (PBS; VWR) for 20 min and then washed twice with PBS. Next, the cells were permeabilized by applying a 0.1% v/v Triton-X100 (Sigma-Aldrich) solution in PBS for 30 min, followed by washing with PBS and a step for blocking nonspecific binding using a casein-based blocking solution (Thermo Fisher Scientific, CAS-Block Histochemical Reagent). Finally, cell nuclei and cytoskeletal F-actin were stained overnight with 4′,6-diamidino-2-phenylindole (DAPI) dihydrochloride (Sigma-Aldrich) and Alexa Fluor 568 phalloidin (Thermo Fisher Scientific, Invitrogen), respectively, both at a dilution of 1:200. The samples were imaged using a confocal laser scanning fluorescence microscope (Leica Microsystems, TCS SP8 STED) by acquiring image series/stacks of 2 μm-thick slices.

### 2.8 Statistical Analysis

For Figure 3B, the experiment was performed with *N* = 2 bioreactors and with *n* = 6 spheroids per bioreactor. The results in this figure are presented as the mean value ± standard deviation. For Figures 3E,F and 5D,E, for each of two bioreactors, the measurements for 0 h on the time axis of the diagrams were at performed at 0, 1, 2, 3, 4, 5, 6, 7, 8, and 9 h and averaged, for 10 h on the time axis of the diagrams at 10, 11, 12, 13, 14, 15, 16, 17, 18, and 19 h and averaged, ···, and for 90 h on the time axis of the diagrams at 90, 91, 92, 93, 94, 95, and 96 h and averaged, respectively. For Figure 4D,E, for each of two bioreactors, the measurements for 0 h on the time axis of the diagrams were performed at 0, 1, 2, 3, 4, 5, 6, 7, 8, and 9 h and averaged, for 10 h on the time axis of the diagrams at 10, 11, 12, 13, 14, 15, 16, 17, 18, and 19 h and averaged, ···, and for 40 h on the time axis of the diagrams at 40, 41, 42, 43, 44, 45, 46, 47, and 48 h and averaged. For Figure S1C,D, the analyses were performed with binning intervals of 50 and 1000 μm^2^ and *n* = 108 Ti and *n* = 82 HA particles, respectively. For Figure S3, the experiments were performed with *N* = 2 bioreactors. The results in this figure are presented as the mean value ± standard deviation.

## 3 Results and Discussion

### 3.1 Characterization of the Bioreactor

The microfluidic bioreactor was developed to monitor cell cultures in real time using EIS analysis, along with the cells’ visual inspection, throughout the culture. This was enabled by the selection of materials for different layers of the bioreactor. The housing of the bioreactor was made from PMMA, a widely used, optically transparent polymer, with good biocompatibility and suitable for cost-effective processing.^33^ The electrodes were made from ITO, transmitting 80 to 90% of the visible light,^34^ which, together with the low resistivity of ITO, makes it one of the most widely used materials for the fabrication of transparent electrodes. The microwells formed from ion track-etched PC films, see Section 3.2., also presented good optical properties. Assembled under sterile conditions, the bioreactor with its PDMS gaskets was found to be leak-free during the culture experiments.

### 3.2 Characterization of Microwell Arrays

The thermoformed microwell arrays consisted of 30 circular microwells hexagonally arranged in 5 rows with 6 wells each (Figure 2A). The microwells had a maximum outer diameter of 542.12 ± 3.36 μm and a maximum inner depth of 181.12 ± 28.59 μm (Figure 2B). Since the stretching of the porous film during the thermoforming process enlarges the size of its pores,^29^ the pore size of the film after thermoforming was measured (Figure 2C). The pore diameter of the film after thermoforming was 1.07 ± 0.17 μm, which corresponds to a 2.68-fold increase. Porous microwells were selected to allow the transfer of ions between the compartments of the microfluidic bioreactor through the microwells’ side walls and bottoms during the EIS measurement. The use of porous films in the context of EIS measurements of the cell microenvironment has been shown in previous studies.^18,35^ However, to the best of our knowledge, this is the first time that thermoformed porous microwells were applied for such measurements. Thermoformed microwells have been previously used to culture cellular spheroids, as well as hybrid cell-microbiomaterial ones, making use of the physical confinement of the cells inside the wells.^12^ The use of highly transparent films makes the regularly arranged microwells compatible with automated microscopic analysis.^36^ The setup used in this study enabled simultaneous monitoring of cell behavior by EIS and light microscopy imaging.

**Figure 2:**
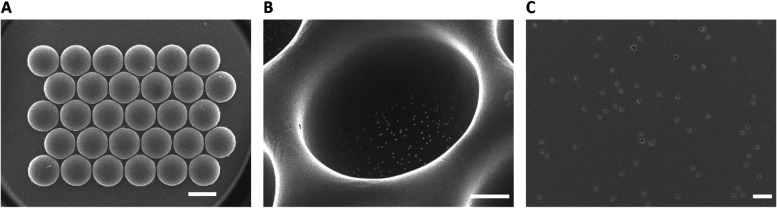
Porous microwell arrays. SEM micrographs of (arrays of) porous microwells at various magnifications. (A) Back view of a microwell array. The scale bar represents 500 μm. (B) 30°-tilted close-up of a single microwell. The scale bar represents 100 μm. (C) Zoomed-in image of the pores in the bottom region of a microwell. The scale bar represents 10 μm.

### 3.3 Spheroid Culture and EIS Measurements

The formation and culture of hMSC spheroids inside the microfluidic bioreactor were assessed over the course of 96 h. Within 2 h after seeding, the hMSCs appeared condensed at the bottom of the microwells, starting to aggregate (Figure 3A). The spheroids formed within the first 24 h of culture, after which they appeared to undergo further compaction for the remaining 72 h of culture. This observation was confirmed by the quantification of the projected area of the spheroids by image analysis (Figure 3B), showing a significantly strong decrease of the spheroid area between 2 and 24 h, followed by a gradual decrease up to 96 h. The observed compaction of hMSC spheroids has been reported in multiple studies^12,37,38^ and is often attributed to the hMSCs’ lack of proliferation and their apoptosis^37,38^ or even to autophagy in the spheroids.^39^

**Figure 3:**
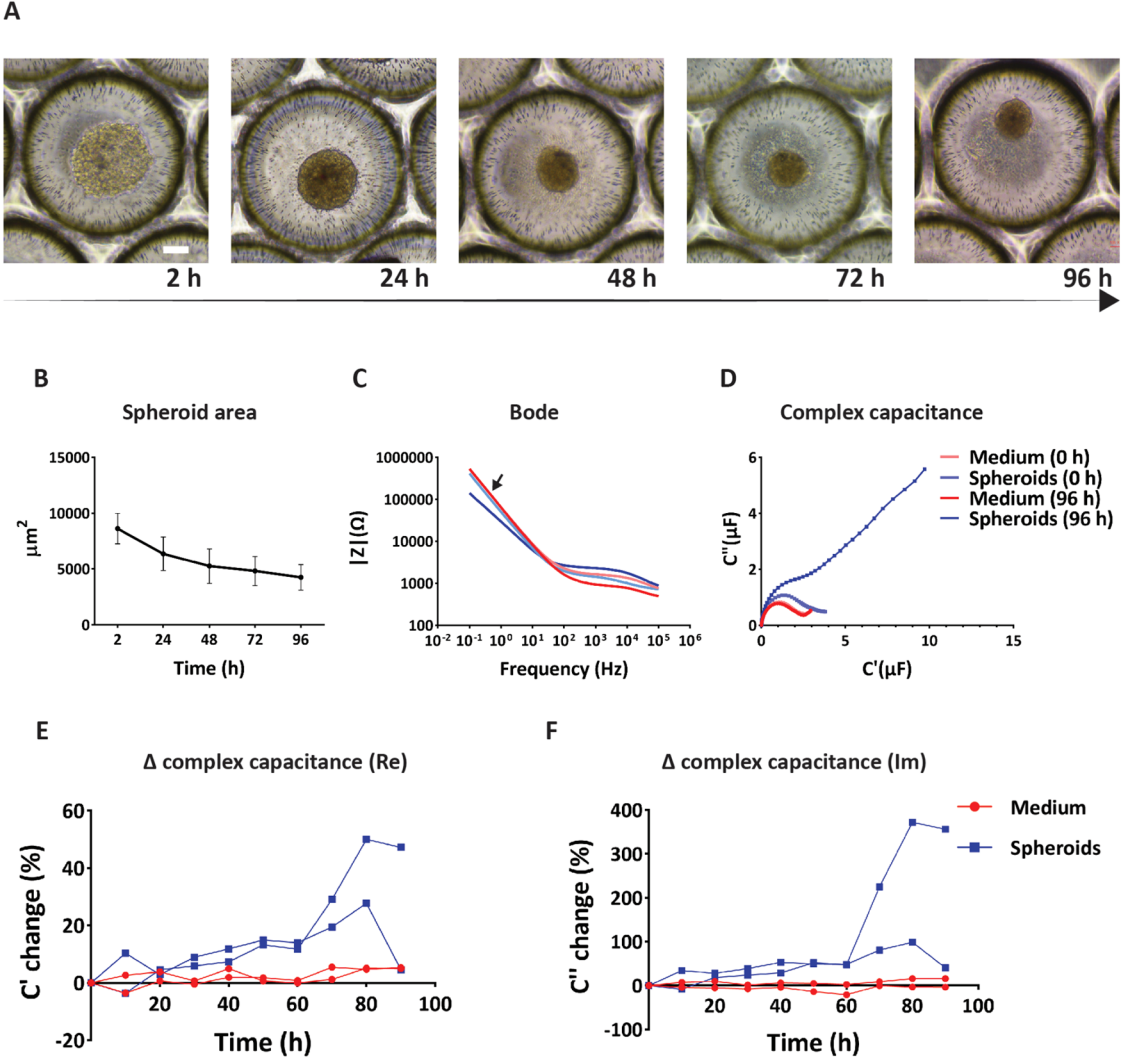
Culture of spheroids in the microfluidic bioreactor. (A) Bright-field microscopy images of (hMSCs and) hMSC spheroids in the microwells inside the bioreactor after 2, 24, 48, 72, and 96 h of culture. The scale bar represents 100 μm and applies to all images. (B) Assessment of hMSC spheroid area after 2, 24, 48, 72, and 96 h of culture in the bioreactor. (C) Bode plot of the magnitude of the impedance signal for hMSC spheroids and for only cell culture medium (for one of two bioreactors) at 0 and 96 h. The black arrow indicates the selected frequency of 1 Hz, where a difference between the spectra for hMSC spheroids and for culture medium at 96 h was observed, a phenomenon starting roughly below 10 Hz. The legend of subfigure (D) applies also to this subfigure. (D) Complex capacitance Nyquist plot for hMSC spheroids and for cell culture medium only (for one of two bioreactors) at a frequency of 1 Hz at 0 and 96 h. (E) Relative changes of the real part of the complex capacitance compared to its value at 0 h over the course of 90 + 6 h (see also Section 2.8) for hMSC spheroids and for culture medium only at a frequency of 1 Hz. The legend of subfigure (F) applies also to this subfigure. (F) Relative changes of the imaginary part of the complex capacitance compared to its value at 0 h over the course of 90 + 6 h (see also Section 2.8) for hMSC spheroids and for culture medium at a frequency of 1 Hz.

Concerning the EIS measurements, first, preliminary data were recorded for a reference condition in which only the cell culture medium was present in the bioreactor, and for the condition in which (also) hMSC spheroids were there. The goal of this initial analysis was to identify the range of frequencies in which complex resistance/impedance signals would indicate the presence of spheroids. Electrical impedance spectra were recorded every 24 h for a total of 96 h. Then, Bode plots of the magnitude of the impedance were created and compared under the two conditions. Bode diagrams at 0 h did not show obvious differences in magnitude between the signals recorded for the condition with and without spheroids (Figure 3C, light blue and light red graphs, respectively). After 96 h of culture, the Bode diagrams of the spheroid and nonspheroid conditions were different at frequencies roughly below 10 Hz (e.g., at 1 Hz) and above 100 Hz (Figure 3C, blue and red graphs, respectively). In this frequency range, a lower impedance signal was observed for spheroids as compared to the only culture medium condition. A similar finding was reported in a study where measurements were performed on human hepatoma and epithelial carcinoma cells in Matrigel.^40^ It was suggested that this difference was due to an increase in the conductivity of the system through neighboring cells, whereby gap junctions act as channels that facilitate the flow of electrical currents. Our result indicated that the setup could sense the presence of spheroids. Based on these preliminary data, and as lower frequencies better describe the electrical properties of cells in medium,^41^ 1 Hz (Figure 3C, black arrow) was selected as the frequency for further analysis.

The electrical behavior of cells has been frequently modeled using an equivalent circuit. Single cells—and, as an attempt at extrapolation, also human tissues—are commonly described by a circuit that represents the bioimpedance of a cell as a capacitor corresponding to the capacitance of the cell membrane (*C*_m_), in series with a resistor representing the intracellular resistance (*R*_i_), and this series connection in parallel with another resistor representing the extracellular resistance (*R*_e_).^42^ Considering this model, the electrical equivalent circuit of a spheroid composed solely of cells within our sensing bioreactor is represented in Figure S2. The proposed equivalent circuit describes the electrochemical behavior of a spheroid within its surrounding cell medium, which mostly acts as a resistor at low frequencies,^43^ and hosting porous microwells from poorly conductive PC.^44^ *C*_m_ has an important contribution at high frequencies as it describes cell membrane charge and discharge phenomena.^45^ By plotting the complex capacitance, which describes the capability of a system to store electric charge, differences between the only medium condition and the hMSC seeded in the microwells were visible from the first measurement (Figure 3D; at 0 h; light red and light blue graphs, with the first being largely covered by the red graph), and they became larger over the course of the experiment (Figure 3D; at 96 h; red and blue graphs). A complex capacitance plot is characterized by two regions, a high-frequency Ohmic region on the left of the plot and a Faradaic region for lower frequencies on the right.^46^ The complex capacitance plot of the reference condition remained constant over time and, at both time points, showed a minimal Faradaic charge-storing region, illustrated by the small tails after the pronounced bumps in the Ohmic region. This outcome was expected, as the medium does not store electric charge and hence does not behave as a capacitor. In contrast, under the spheroid condition, the complex capacitance plot appeared rather different. After 96 h of culture, the Faradaic region had clearly higher values compared to the reference condition, as well as compared to 0 h. This means that the spheroids exhibited more evident capacitor-like behavior, which increased over time.

Based on these considerations, the complex capacitance was separated into its real part C′ (Figures 3E and S3A) and imaginary part C″ (Figures 3F and S3B) and analyzed over time at the selected frequency of 1 Hz. The graphs were normalized to the signal at 0 h, the time point of the first EIS measurement. This normalization has been done for EIS analysis of tissues elsewhere.^47^ An obvious difference between the graphs of the reference condition with only cell culture medium and the condition with spheroids was apparent in both the real and imaginary parts of the signal. For the reference condition, no obvious temporal changes were observed in the capacitance plot of either the real or the imaginary part. For the spheroids, the real part of the complex capacitance plot showed an initial adjustment phase over the course of the first 20 h, without a clear trend in the capacitance signal (Figure 3E). Next, a steady increase of the signal value was observed up to 80 h into the measurement. At that point, a different behavior was observed in the two replicates of the spheroid conditions, with one of them exhibiting an abrupt drop of the signal and the other showing a comparatively smaller reduction of the signal. The imaginary part of the complex capacitance also showed a variation between the signals of the two spheroid replicates over time, similarly to the real part (Figure 3F). In particular, the trend of the signal in the two spheroid replicates almost overlapped up to 60 h. Between 60 and 80 h, the signals diverged, with one of them showing a small change and the other a considerably bigger change over time. At 80 h, a drop in the intensity of the signal was observed for both replicates, similar to the real part.

In the case of measurements on single cells, particularly at low frequencies, the application of a current polarizes the cell membrane, and the ions contained in the cytoplasm align to the applied current. This creates a charge separation at the cell membrane.^48^ The capacitance of cells depends on the cell type, metabolism, and growth phase, but only cells with an intact cell membrane can store charge.^49,50^ High capacitance values of cell spheroids may indicate cell cohesion and tightness within the spheroids. Although further experiments are required to obtain more conclusive results and to be able to explain the underlying biological mechanisms, with the results presented in this section, we provide the first proof of concept of the suitability of the bioreactor for monitoring the electrical properties of hMSC spheroids over time by means of EIS.

### 3.4 EIS Analysis of hMSC Spheroids and 2D Culture

After we established the microfluidic bioreactor setup as a suitable environment to perform EIS measurements on hMSC spheroids, we proceeded to evaluate differences in the electrochemical behavior of the spheroids in comparison with a 2D hMSC culture. In the latter case, as mentioned in Section 2.4, the microwells were not coated with Pluronic. For both the 2D and 3D conditions, the same number of cells was seeded into the culture chamber of the bioreactor. The hMSCs, as spheroids or in 2D, were cultured for 48 h, after which they were fixed and stained for cell nuclei and F-actin (Figure 4A). Below roughly 10 Hz, the Bode plots did not show any obvious changes over the 48-h analysis for either the 2D or the spheroid condition (Figure 4B). However, for frequencies above roughly 100 Hz, the impedance magnitude of the hMSC spheroid condition was higher than the one of the 2D hMSC culture. Regarding their resistive behavior, the already forming spheroid extracellular matrix (ECM) might have presented a resistance to the ion current, which can explain this result.^49^ The complex capacitances of spheroids and 2D cultures appeared rather different, with the exception of the very first steep slope common to both graphs (Figure 4C). The spheroids showed a higher Ohmic as well as Faradaic region of the complex capacitance plot compared to the 2D hMSC culture. In line with the differences between the spheroids and the medium reference, see Section 3.3., the higher Faradaic region in the spheroid condition compared to the 2D hMSC culture can be attributed to the storage of electrical charge in the spheroids. Next, we analyzed the complex capacitance of hMSC spheroids and 2D cultures by separately plotting the real part (Figures 4D and S3C) and the imaginary part (Figures 4E and S3D). For the real part, after an initial adjustment phase within the first 20 h of the measurement, the spheroids showed an increase in the capacitance signal over time. The hMSCs cultured in 2D also showed an adjustment phase within the first 20 h; then, however, the capacitance signal remained roughly constant until the end of the measurement. The adjustment phase may correspond to the aggregation and compaction phases for the spheroids and to the attachment and adhesion phases in the case of the 2D culture. For the imaginary part, both the spheroids and 2D culture showed a trend similar to that of the real part of the signal. As already shown in Section 3.3, this confirmed the suggested capability of the spheroids to store electrical charge at the surface of the cell membrane as well as within the intercellular gaps where the ECM is deposited. We observed larger differences in the EIS signals among the tested replicates. For the 2D culture data, the number of cells covering and obstructing the pores of the microwells may have varied, potentially causing some signal variability.

**Figure 4:**
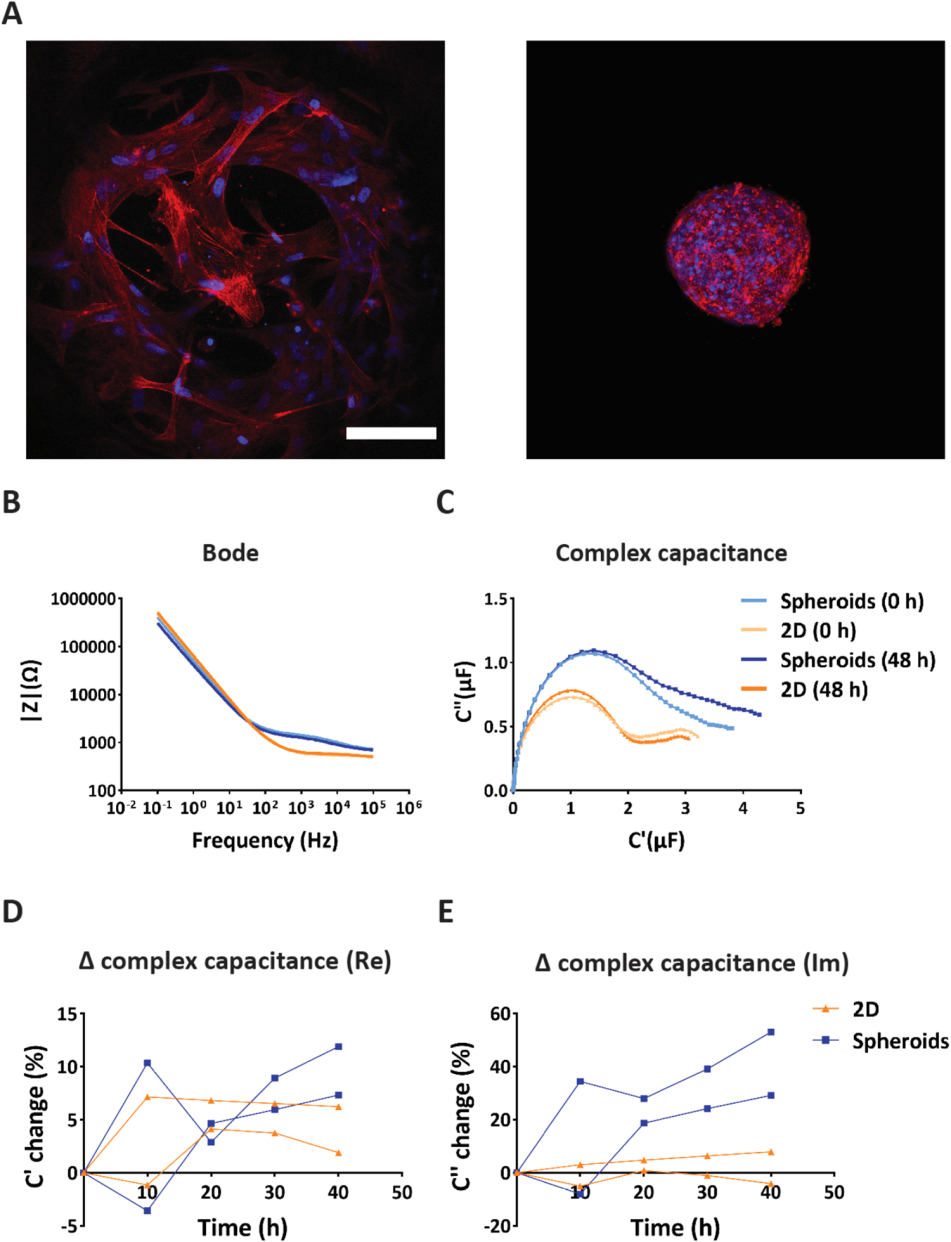
Spheroid versus 2D hMSC culture inside the microfluidic bioreactor. (A) Maximum intensity projection of confocal fluorescence microscopy images of hMSCs cultured in 2D (left) and as spheroids (right) in the microwells inside the bioreactor after 48 h of culture. The cells were stained for their cytoskeletons (red) and nuclei (blue). The scale bar represents 100 μm and applies to both images. (B) Bode plot of the magnitude of the impedance signal for hMSC spheroids and 2D culture (for one of two bioreactors) at 0 and 48 h. The legend of subfigure (C) applies also to this subfigure. (C) Complex capacitance Nyquist plot for hMSC spheroids and 2D culture (for one of two bioreactors) at a frequency of 1 Hz at 0 and 48 h. (D) Relative changes of the real part of the complex capacitance compared to its value at 0 h over the course of 40 + 8 h (see also Section 2.8) for hMSC spheroids and 2D culture at a frequency of 1 Hz. The legend of subfigure (E) applies also to this subfigure. (E) Relative changes of the imaginary part of the complex capacitance compared to its value at 0 h over the course of 40 + 8 h (see also Section 2.8) for hMSC spheroids and 2D culture at a frequency of 1 Hz.

### 3.5 EIS Analysis of Hybrid Cell-Microbiomaterial Spheroids

We also tested the proposed setup for its ability to monitor the formation and culture of hybrid cell-microbiomaterial spheroids. Coaggregated with cells, microbiomaterials, as introduced by us,^51,52^ can take on the role of spacers reducing the amount of needed patient-derived and expanded cells in implantable constructs, microscaffolds that are remodelable from the very beginning on and without necessary degradation and erosion, cell-instructive miniscaffolds, 3D-assessable micromaterials in early biomaterials R&D,^12^ biomimetic (e.g., collagen-based) ECM in *in vitro* model tissues and tissues for implantation, etc. Two reference materials, Ti and HA, in the shape of microparticles, were selected. Apart from their chemistry, they also differ in their porosity and surface roughness, plausibly causing different electrochemical behavior. Previously, EIS has been used to assess the surface porosity of Ti implants^53^ and proposed as a method to study biomaterial interfaces.^54^ In our study, hMSCs and biomaterial microparticles were coseeded onto the microwell array inside the chamber of the microfluidic bioreactor. Similarly to the hMSC-only spheroids (Figure 3A), hMSC-Ti and hMSC-HA spheroids formed within the first 24 h and compacted over time (Figure 5A). Similar to the observations in Section 3.3, the Bode plots showed differences in the low frequency range for the spectra of the hMSC, hMSC-Ti, and hMSC-HA spheroids after 48 h of measurement (Figure 5B), which is why the 1 Hz frequency was chosen also here to conduct further analysis. The complex capacitance plot of the different hybrid spheroids underlined differences in their capacitive behavior (Figure 5C). In particular, a major increase in the signal in the Faradaic region for both the hMSC and hMSC-HA spheroids was visible at 96 h (blue and purple graphs, respectively), while the hMSC-Ti spheroids showed a less pronounced increase (green graph). As mentioned in Sections 3.3 and 3.4, the hMSC spheroids exhibit an increase in capacitance as they compact over time. In the case of hMSC-HA spheroids, the highly microporous microparticles from HA (Figure S1B),^55^ previously shown to act as an anionic conductor,^56,57^ may have contributed to the observed increase in the complex capacitance as the surface of HA acts as an ion reservoir when in an electrolyte solution.^46^ In contrast to hMSC-HA spheroids, hMSC-Ti spheroids did not show the same behavior, most likely because the Ti microparticles presented a smooth and nonporous surface (Figure S1A), which could not act as an ion reservoir. These initial results suggest that the EIS analysis may be suitable to distinguish the presence of different materials in the spheroids. In terms of the variation of the complex capacitance over time, we evaluated the changes in the real and imaginary parts of the signal over the course of 96 h (Figures 5D,E, and S3E,F, respectively). For both the real and imaginary parts, and in both hybrid spheroids as well as in the hMSC-only spheroids, no obvious variations in the complex capacitance plots were observed during the first roughly 40 h. Afterward, the hMSC spheroids showed an increase in both their C′ and C″ values, before there was a decline at around 80 h. For both the hMSC-Ti and hMSC-HA spheroids, we observed larger differences in the EIS signals between the tested replicates. This may be because of variations in spheroid size, shape, and morphology, as well as due to variations in the amount and (average) size of microparticles dispensed into each microwell and aggregated into each spheroid. The effect of the properties and amount of microbiomaterials was also investigated in a previous study from our group.^12^ Among others, optimization of particle production, selection, and seeding procedures might be required to produce more conclusive results.

**Figure 5:**
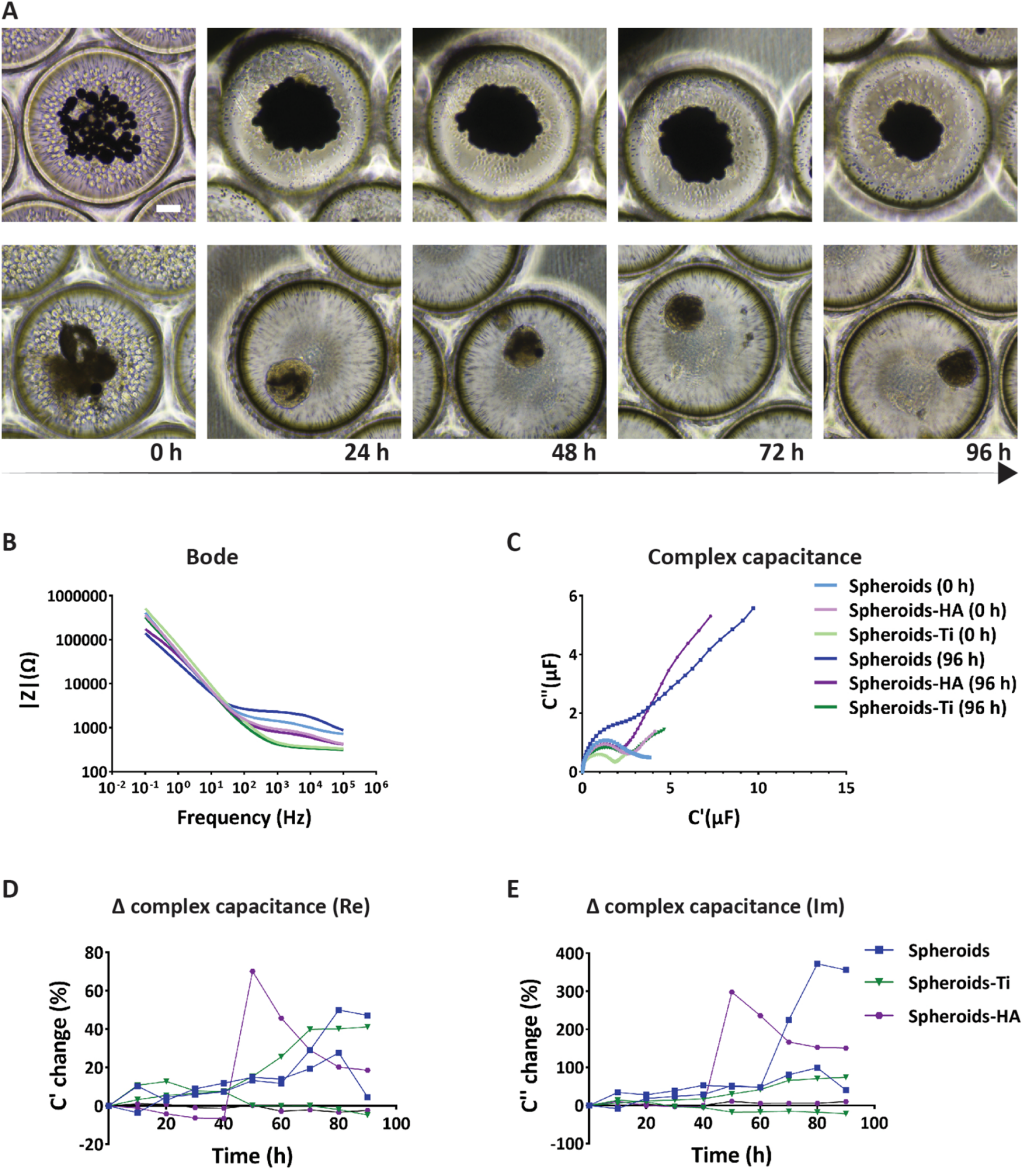
Cell-microbiomaterial spheroids inside the microfluidic bioreactor. (A) Bright-field microscopy images of hMSC-Ti (top row) and hMSC-HA (bottom row) spheroids in microwells inside the bioreactor after 0, 24, 48, 72, and 96 h of culture. The scale bar represents 100 μm and applies to all images. (B) Bode plot of the magnitude of the impedance signal for hMSC, hMSC-Ti, and hMSC-HA spheroids at 0 and 96 h. The legend of subfigure (C) applies also to this subfigure. (C) Complex capacitance Nyquist plot for hMSC, hMSC-Ti, and hMSC-HA spheroids (for one of two bioreactors) at a frequency of 1 Hz at 0 and 96 h. (D) Relative changes of the real part of the complex capacitance compared to its value at 0 h over the course of 90 + 6 h (see also Section 2.8) for hMSC, hMSC-Ti, and hMSC-HA spheroids at a frequency of 1 Hz. The legend of subfigure (E) applies also to this subfigure. (E) Relative changes of the imaginary part of the complex capacitance compared to its value at 0 h over the course of 90 + 6 h (see also Section 2.8) for hMSC, hMSC-Ti, and hMSC-HA spheroids at a frequency of 1 Hz.

## 4 Conclusions and Outlook

In this study, we propose a microfluidic bioreactor to culture and nondestructively monitor hMSC spheroids over time using EIS. For this, an array of porous microwells thermoformed into track-etched films and a pair of sensing electrodes from transparent ITO were integrated into the chamber of the bioreactor. To measure the spheroid’s electrical properties, the electrodes were connected to an FRA, with a multiplexer in between to enable the operation of two bioreactors at the FRA at the same time. We found differences between the complex resistance/impedance and/or capacitance data of a reference condition without cells, a 2D hMSC culture, hMSC spheroids, and hybrid spheroids aggregated from hMSCs and Ti or HA microparticles. We also found differences between different culture durations. These results suggest that our device can sense the presence and spatial arrangement of cells and microbio materials as a function of time.

Follow-up work should include further characterization and optimization of the whole setup. The latter particularly concerns improvements in cell seeding as well as in microparticle fabrication, selection, and dispensing procedures, which can be assumed to be a major cause of signal variability. Future applications of the system may include the characterization of the behavior of (hybrid cell-microbiomaterial) spheroids in terms of viability, metabolic activity, proliferation, apoptosis, and mineralization, to name a few. In this context, the EIS spectra data would be validated by comparison with endpoint functional assay readouts, such as live–dead staining, resazurin staining, EdU cell proliferation assay, lactate dehydrogenase assay, and Alizarin Red staining, respectively. Subsequently, applications of this technology could also extend to small compound and drug testing, to make it an effective method for assessing drug safety and dosage in real time. An interesting further development could be the modification of the electrodes and their switching interface with the FRA to enable single-microwell-resolved EIS measurements.

## Supplementary Material



## Notes

Appearance and size distribution of Ti and HA microparticles; electrical equivalent circuit model of a spheroid within a porous PC microwell; and averaged time courses of the real and imaginary parts of the complex capacitances from Figures 3–5 (PDF)

## Abbreviations

2D: two-dimensional
3D: three-dimensional
ECM: extracellular matrix
EdU: 5-ethynyl-2′-deoxyuridine
EIS: electr­(ochem)­ical impedance spectroscopy
FRA: frequency response analyzer
HA: hydroxyapatite
hMSC: human mesenchymal stem cell
ITO: indium tin oxide
PBS: phosphate-buffered saline
PC: polycarbonate
PDMS: poly­(dimethylsiloxane)
PET: poly­(ethylene terephthalate)
PMMA: poly­(methyl methacrylate)
PP: polypropylene
SEM: scanning electron microscopy
STED: stimulated emission depletion
Ti: titanium
