# Supplementary material for: Real-Time Monitoring of the Formation and Culture of Hybrid Cell-Microbiomaterial Spheroids Using Non-Faradaic Electrical Impedance Spectroscopy
Source: ACS Biomater Sci Eng. 2025 Sep 18;11(10):6107–18. doi: 10.1021/acsbiomaterials.5c00402 (PMC12522083; doi:10.1021/acsbiomaterials.5c00402)
Supplement: Supplementary file 1 [file ab5c00402_si_001.pdf]

Supporting Information

Real-Time Monitoring of the Formation and Culture of Hybrid Cell-Microbiomaterial Spheroids Using Non-Faradaic Electrical Impedance Spectroscopy

Maria G. Fois, Seppe Bormans, Thijs Vandenryt, Alexander P. M. Guttenplan, Yousra Alaoui Selsouli, Clemens van Blitterswijk, Zeinab Tahmasebi Birgani, Stefan Giselbrecht, Pamela Habibović, Ronald Thoelen, and Roman K. Truckenmüller

4 pages, 3 figures

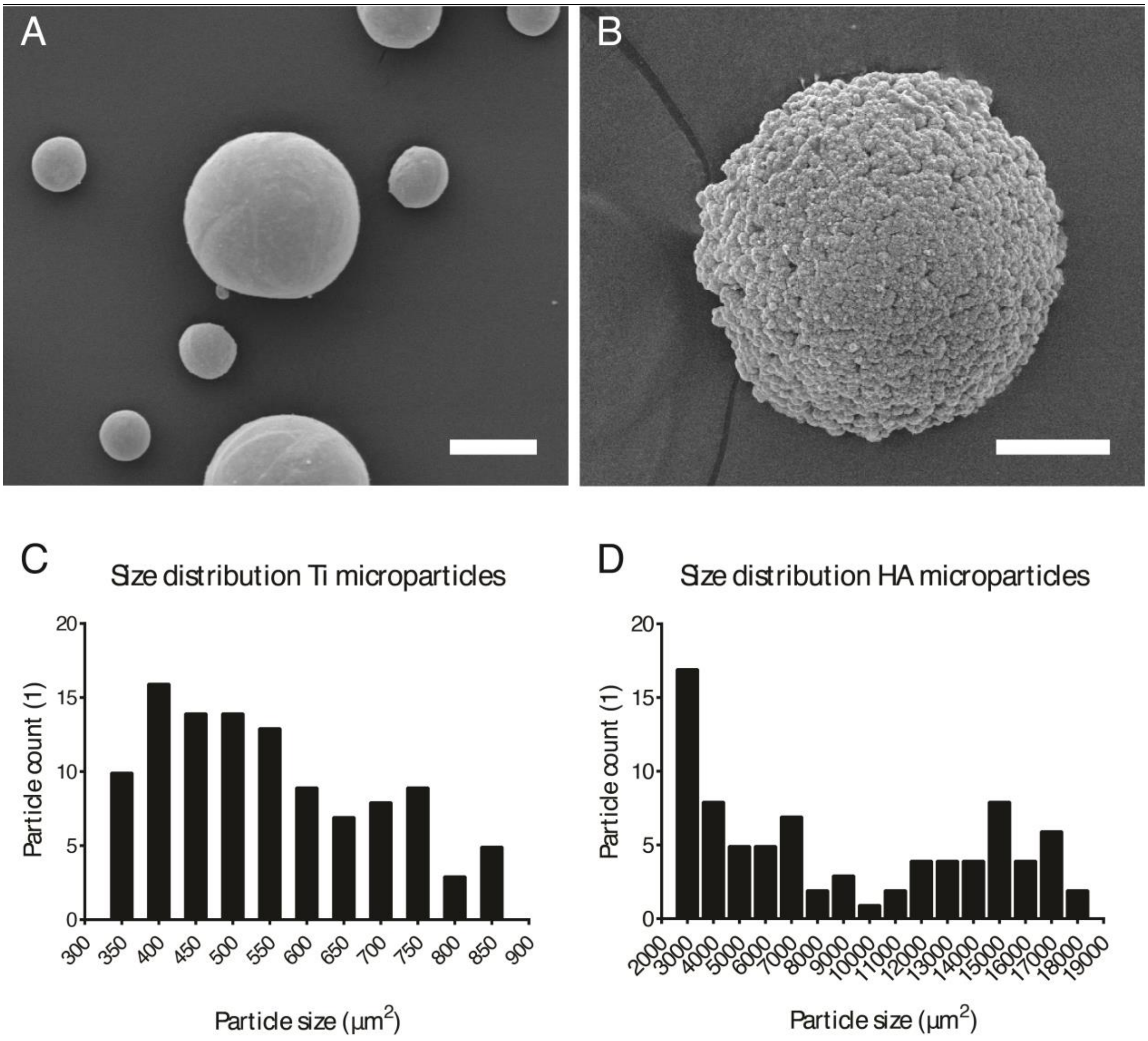

Figure S1. Appearance and size distribution of Ti and HA microparticles. SEM images of (A) representative Ti microparticles and their smooth surface and (B) a representative HA microparticle and its microporous structure. The scale bars represent 20 and 50 μm, respectively. Size distribution of (C) Ti and (D) HA microparticles.

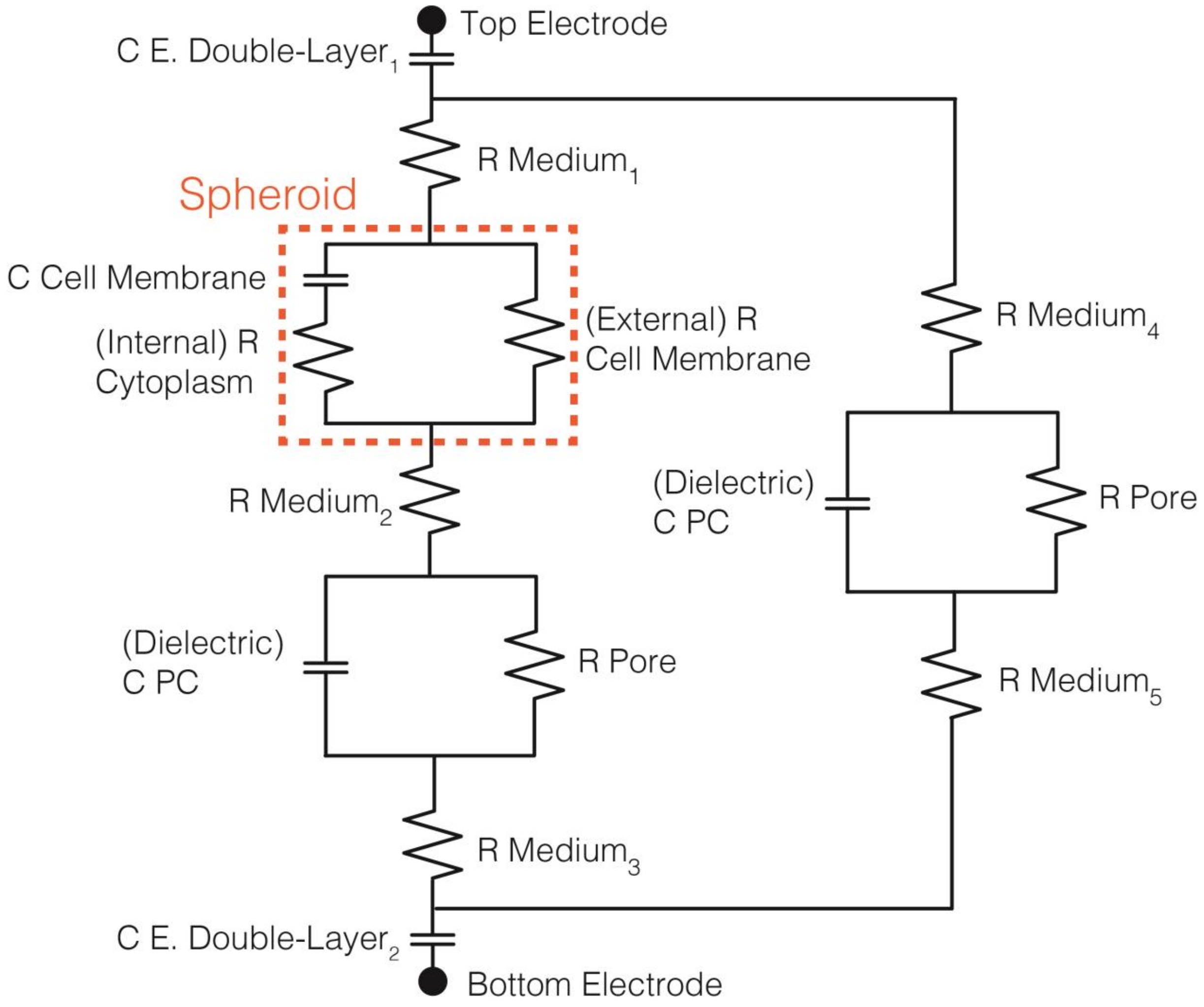

Figure S2. Electrical equivalent circuit model of a spheroid within a porous PC microwell. At the interface between the two electrodes and the culture medium as an electrolyte solution, double-layer capacitances (C E. Double-$Layer_1$ and C E. Double-$Layer_2$) form (as in non-Faradaic systems). Between the top and bottom electrodes, the ion current first goes through the medium, represented as a resistive element (R $Medium_1$). Then, it finds the spheroid, which is modeled as a capacitive element – representing the charge-storing cell membrane – (C Cell Membrane) in series with the internal resistance – i.e., the resistance of the cytoplasm – of the cells forming the spheroid ((Internal) R Cytoplasm). This series of elements is in parallel with the external resistance of the cells forming the spheroid ((External) R Cell Membrane). Next, the current goes through some

medium entrapped between the spheroid and the microwell (R Medium$_2$). Then, it goes through (the medium in) the pores in the bottom of the microwell (R Pore) or, in parallel, the PC film material around the pores, which – due to its poor conductivity – is modeled as a small dielectric capacitance ((Dielectric) C PC). Finally, the current again goes through medium (R Medium$_3$). Alternatively, the current bypasses the spheroid through the pores in the side walls of the microwell, which are not obstructed by the presence of the spheroid, or the material around them (R Medium$_4$, R Pore, (Dielectric) C PC, and R Medium$_5$). This electrical bypass is in parallel with the path through the spheroid.

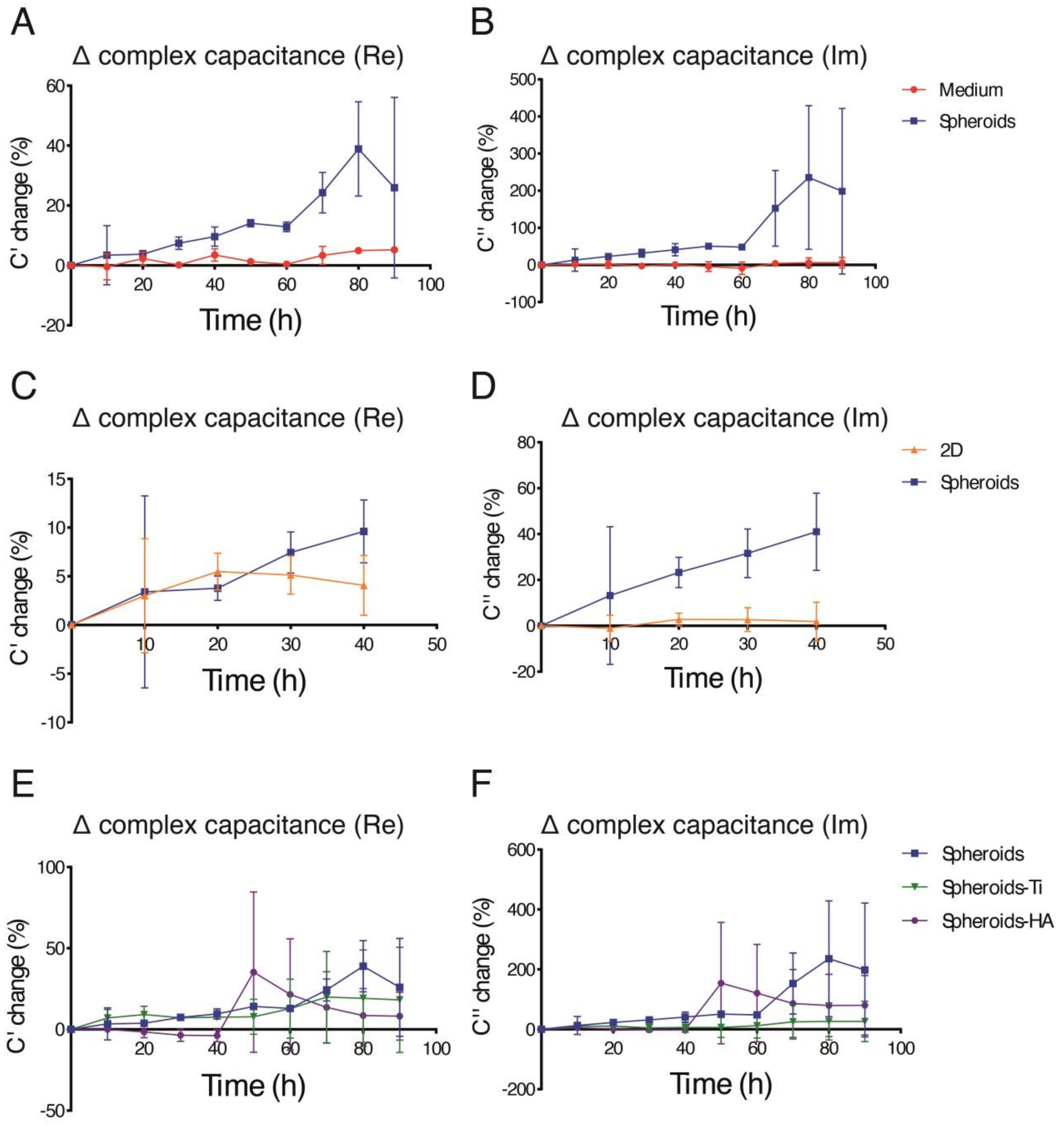

Figure S3. Averaged time courses of the real and imaginary parts of the complex capacitances from Figures 3-5. Figures derived from (A and B) **Figures 3**E,F, (C and D) **4**D,E, and (E and F) **5**D,E, respectively, with C′ and C″ as the average of the signals from the replicates.
